# Diaqua­iodido(2,3,5,6-tetra-2-pyridyl­pyrazine-κ^3^ *N* ^2^,*N* ^1^,*N* ^6^)manganese(II) iodide monohydrate

**DOI:** 10.1107/S1600536811035434

**Published:** 2011-09-14

**Authors:** Kwang Ha

**Affiliations:** a School of Applied Chemical Engineering, The Research Institute of Catalysis, Chonnam National University, Gwangju 500-757, Republic of Korea

## Abstract

The asymmetric unit of the title compound, [MnI(C_24_H_16_N_6_)(H_2_O)_2_]I·H_2_O, consists of a cationic Mn^II^ complex, an I^−^ anion and a solvent water mol­ecule. In the complex, the Mn^II^ ion is six-coordinated in a considerably distorted octa­hedral environment defined by three N atoms of the tridentate 2,3,5,6-tetra-2-pyridyl­pyrazine (tppz) ligand, one I^−^ anion and two O atoms of two water ligands. The dihedral angles between the pyridyl rings [maximum deviation = 0.034 (6) Å] and their carrier pyrazine ring [maximum deviation = 0.020 (6) Å] are 26.5 (2) and 27.0 (2)° for the coordinated pyridyl rings, and 51.3 (3) and 43.2 (2)° for the uncoordinated pyridyl rings. Inter­molecular O—H⋯O, O—H⋯N and O—H⋯I hydrogen bonds stabilize the crystal structure.

## Related literature

For the crystal structures of mono- and dinuclear Mn^II^ complexes with tppz, see: Callejo *et al.* (2009 ▶); Ha (2011 ▶).
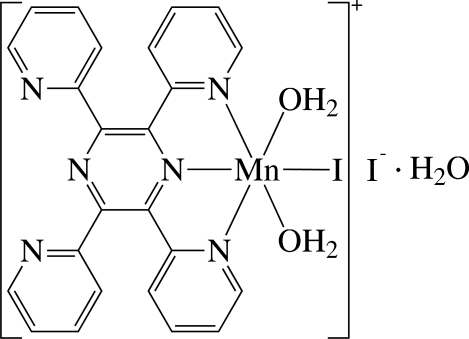


## Experimental

### 

#### Crystal data

[MnI(C_24_H_16_N_6_)(H_2_O)_2_]I·H_2_O
*M* *_r_* = 751.22
Monoclinic, 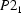

*a* = 7.977 (3) Å
*b* = 13.880 (5) Å
*c* = 12.134 (4) Å
β = 97.944 (7)°
*V* = 1330.5 (8) Å^3^
*Z* = 2
Mo *K*α radiation
μ = 2.85 mm^−1^
*T* = 200 K
0.28 × 0.20 × 0.12 mm

#### Data collection

Bruker SMART 1000 CCD diffractometer
Absorption correction: multi-scan (*SADABS*; Bruker, 2000 ▶) *T*_min_ = 0.828, *T*_max_ = 1.000
9685 measured reflections
5463 independent reflections
4463 reflections with *I* > 2σ(*I*)
*R*_int_ = 0.030

#### Refinement

*R*[*F* ^2^ > 2σ(*F* ^2^)] = 0.040
*wR*(*F* ^2^) = 0.115
*S* = 1.23
5463 reflections
325 parameters
1 restraint
H-atom parameters constrained
Δρ_max_ = 1.06 e Å^−3^
Δρ_min_ = −1.41 e Å^−3^
Absolute structure: Flack (1983 ▶); 2033 Friedel pairs
Flack parameter: −0.01 (4)

### 

Data collection: *SMART* (Bruker, 2000 ▶); cell refinement: *SAINT* (Bruker, 2000 ▶); data reduction: *SAINT*; program(s) used to solve structure: *SHELXS97* (Sheldrick, 2008 ▶); program(s) used to refine structure: *SHELXL97* (Sheldrick, 2008 ▶); molecular graphics: *ORTEP-3* (Farrugia, 1997 ▶) and *PLATON* (Spek, 2009 ▶); software used to prepare material for publication: *SHELXL97*.

## Supplementary Material

Crystal structure: contains datablock(s) global, I. DOI: 10.1107/S1600536811035434/zq2121sup1.cif

Structure factors: contains datablock(s) I. DOI: 10.1107/S1600536811035434/zq2121Isup2.hkl

Additional supplementary materials:  crystallographic information; 3D view; checkCIF report

## Figures and Tables

**Table:** 

Mn1—O1	2.163 (6)
Mn1—O2	2.182 (6)
Mn1—N6	2.245 (7)
Mn1—N1	2.253 (7)
Mn1—N3	2.259 (7)
Mn1—I1	2.7772 (16)

**Table:** 

N6—Mn1—N1	71.7 (3)
N1—Mn1—N3	71.4 (2)

**Table 2:** Hydrogen-bond geometry (Å, °)

*D*—H⋯*A*	*D*—H	H⋯*A*	*D*⋯*A*	*D*—H⋯*A*
O1—H1*A*⋯O3^i^	0.84	1.92	2.726 (10)	160
O1—H1*B*⋯N2^ii^	0.84	2.10	2.890 (10)	156
O2—H2*A*⋯N4^iii^	0.84	2.04	2.876 (10)	174
O2—H2*B*⋯N5^iii^	0.84	2.03	2.834 (10)	160
O3—H3*A*⋯I1^iv^	0.84	2.88	3.615 (9)	148
O3—H3*B*⋯I2	0.84	2.83	3.646 (8)	163

Symmetry codes: (i) 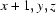; (ii) 
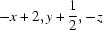
; (iii) 
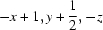
; (iv) 
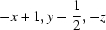
.

**Fig. 1.**
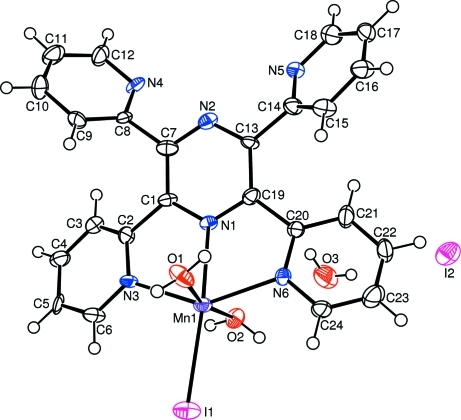
A structure detail of the title compound, with displacement ellipsoids drawn at the 40% probability level for non-H atoms.

**Fig. 2.**
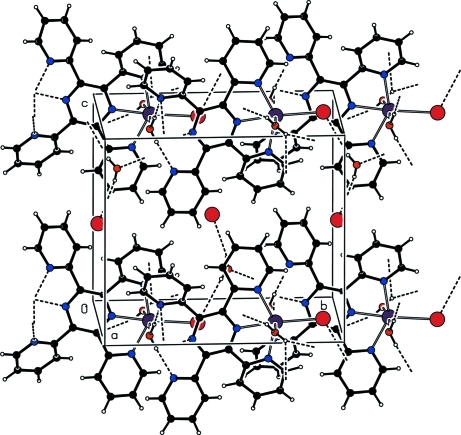
View of the unit-cell contents of the title compound. Hydrogen-bond interactions are drawn with dashed lines.

## supplementary crystallographic information

### Comment

Mono- and dinuclear Mn^II^ complexes of 2,3,5,6-tetra-2-pyridylpyrazine (tppz; C_24_H_16_N_6_) ligand, such as [Mn(C_2_N_3_)(NO_3_)(tppz)(H_2_O)] (Callejo *et al.*, 2009) and [Mn_2_Cl_4_(tppz)_2_] (Ha, 2011), have been investigated previously.

The asymmetric unit of the title compound, [MnI(tppz)(H_2_O)_2_]I.H_2_O, consists of a cationic Mn^II^ complex, an I^-^ anion and a solvent water molecule (Fig. 1). In the complex, the Mn^II^ ion is six-coordinated in a considerably distorted octahedral environment defined by three N atoms of the tridentate tppz ligand, one I^-^ anion and two O atoms of two water ligands. The main contribution to the distortion is the tight N—Mn—N chelating angles (Table 1), which results in non-linear *trans* arrangement of the N3—Mn1—N6 bond [N3—Mn1—N6 = 143.1 (3)°]. The apical O1—Mn1—O2 and I1—Mn1—N1 bond angles are 167.0 (2)° and 173.04 (19)°, respectively. The three Mn—*N*(pyrazine or pyridyl) bond lengths are roughly equivalent and slightly longer than the Mn—O(H_2_O) bonds (Table 1). In the crystal structure, the pyrazine and pyridyl rings are nearly planar [maximum deviation = 0.020 (6) Å for pyrazine and 0.034 (6) Å for pyridyl]. The dihedral angles between the pyridyl rings and their carrier pyrazine ring are 26.5 (2)° and 27.0 (2)° for the coordinated pyridyl rings, and 51.3 (3)° and 43.2 (2)° for the uncoordinated pyridyl rings, respectively. The complex, anion and solvent water molecule are linked by intermolecular O—H···O, O—H···N and O—H···I hydrogen bonds (Fig. 2, Table 2). In addition, the complex displays numerous inter- and intramolecular π-π interactions between adjacent six-membered rings, the shortest ring centroid-centroid distance being 4.032 (5) Å.

### Experimental

To a suspension of 2,3,5,6-tetra-2-pyridylpyrazine (0.1945 g, 0.501 mmol) in acetone (20 ml) was added MnI_2_ (0.1575 g, 0.510 mmol) in acetone (30 ml) and refluxed for 5 h. The formed precipitate was separated by filtration, washed with acetone and dried at 50 °C, to give an orange powder (0.0779 g). Crystals suitable for X-ray analysis were obtained by slow evaporation from a 2-butanone solution.

### Refinement

Carbon-bound H atoms were positioned geometrically and allowed to ride on their respective parent atoms [C—H = 0.95 Å and *U*_iso_(H) = 1.2*U*_eq_(C)]. The H atoms of the water ligands and solvent molecule were located from Fourier difference maps then allowed to ride on their parent O atoms in the final cycles of refinement with O—H = 0.84 Å and *U*_iso_(H) = 1.5 *U*_eq_(O). The highest peak (1.06 e Å^-3^) and the deepest hole (-1.41 e Å^-3^) in the difference Fourier map are located 2.23 Å and 0.80 Å from the I2 atom, respectively. The Flack parameter is -0.01 (4) in the final cycles of refinement.

### Crystal data

**Table:**

[MnI(C_24_H_16_N_6_)(H_2_O)_2_]I·H_2_O	*F*(000) = 726
*M**_r_* = 751.22	*D*_x_ = 1.875 Mg m^−^^3^
Monoclinic, *P*2_1_	Mo *K*α radiation, λ = 0.71073 Å
Hall symbol: P 2yb	Cell parameters from 5752 reflections
*a* = 7.977 (3) Å	θ = 2.6–28.3°
*b* = 13.880 (5) Å	µ = 2.85 mm^−^^1^
*c* = 12.134 (4) Å	*T* = 200 K
β = 97.944 (7)°	Plate, orange
*V* = 1330.5 (8) Å^3^	0.28 × 0.20 × 0.12 mm
*Z* = 2	

### Data collection

**Table:**

Bruker SMART 1000 CCD diffractometer	5463 independent reflections
Radiation source: fine-focus sealed tube	4463 reflections with *I* > 2σ(*I*)
graphite	*R*_int_ = 0.030
φ and ω scans	θ_max_ = 28.4°, θ_min_ = 2.2°
Absorption correction: multi-scan (*SADABS*; Bruker, 2000)	*h* = −10→10
*T*_min_ = 0.828, *T*_max_ = 1.000	*k* = −18→14
9685 measured reflections	*l* = −16→13

### Refinement

**Table:**

Refinement on *F*^2^	Secondary atom site location: difference Fourier map
Least-squares matrix: full	Hydrogen site location: inferred from neighbouring sites
*R*[*F*^2^ > 2σ(*F*^2^)] = 0.040	H-atom parameters constrained
*wR*(*F*^2^) = 0.115	*w* = 1/[σ^2^(*F*_o_^2^) + (0.*P*)^2^ + 7.6642*P*] where *P* = (*F*_o_^2^ + 2*F*_c_^2^)/3
*S* = 1.23	(Δ/σ)_max_ < 0.001
5463 reflections	Δρ_max_ = 1.06 e Å^−^^3^
325 parameters	Δρ_min_ = −1.41 e Å^−^^3^
1 restraint	Absolute structure: Flack (1983); 2033 Friedel pairs
Primary atom site location: structure-invariant direct methods	Flack parameter: −0.01 (4)

### Special details

**Table:**

Geometry. All e.s.d.'s (except the e.s.d. in the dihedral angle between two l.s. planes) are estimated using the full covariance matrix. The cell e.s.d.'s are taken into account individually in the estimation of e.s.d.'s in distances, angles and torsion angles; correlations between e.s.d.'s in cell parameters are only used when they are defined by crystal symmetry. An approximate (isotropic) treatment of cell e.s.d.'s is used for estimating e.s.d.'s involving l.s. planes.
Refinement. Refinement of *F*^2^ against ALL reflections. The weighted *R*-factor *wR* and goodness of fit *S* are based on *F*^2^, conventional *R*-factors *R* are based on *F*, with *F* set to zero for negative *F*^2^. The threshold expression of *F*^2^ > σ(*F*^2^) is used only for calculating *R*-factors(gt) *etc*. and is not relevant to the choice of reflections for refinement. *R*-factors based on *F*^2^ are statistically about twice as large as those based on *F*, and *R*- factors based on ALL data will be even larger.

### Fractional atomic coordinates and isotropic or equivalent isotropic displacement parameters (Å^2^)

**Table:**

	*x*	*y*	*z*	*U*_iso_*/*U*_eq_	
Mn1	0.73505 (17)	0.72431 (9)	0.04312 (11)	0.0215 (3)	
I1	0.72966 (9)	0.92402 (4)	0.05631 (6)	0.03842 (18)	
O1	1.0067 (8)	0.7096 (5)	0.0533 (6)	0.0328 (16)	
H1A	1.0537	0.6728	0.1032	0.049*	
H1B	1.0648	0.7508	0.0246	0.049*	
O2	0.4663 (7)	0.7099 (5)	0.0564 (5)	0.0263 (14)	
H2A	0.4295	0.7353	0.1111	0.039*	
H2B	0.4199	0.7271	−0.0070	0.039*	
N1	0.7180 (9)	0.5642 (5)	0.0129 (6)	0.0187 (15)	
N2	0.7871 (9)	0.3757 (5)	−0.0207 (6)	0.0233 (16)	
N3	0.6873 (9)	0.6981 (5)	−0.1423 (6)	0.0207 (15)	
N4	0.6763 (9)	0.3041 (5)	−0.2331 (6)	0.0247 (17)	
N5	0.7592 (9)	0.2438 (5)	0.1416 (6)	0.0242 (17)	
N6	0.7658 (10)	0.6496 (6)	0.2088 (6)	0.0238 (17)	
C1	0.7137 (11)	0.5310 (6)	−0.0902 (7)	0.0193 (18)	
C2	0.6586 (12)	0.6064 (7)	−0.1792 (7)	0.0230 (19)	
C3	0.5767 (12)	0.5849 (7)	−0.2810 (8)	0.027 (2)	
H3	0.5502	0.5199	−0.3011	0.032*	
C4	0.5320 (12)	0.6590 (7)	−0.3557 (8)	0.028 (2)	
H4	0.4761	0.6454	−0.4284	0.034*	
C5	0.5696 (11)	0.7531 (7)	−0.3233 (6)	0.0245 (19)	
H5	0.5455	0.8049	−0.3743	0.029*	
C6	0.6431 (12)	0.7699 (7)	−0.2151 (8)	0.027 (2)	
H6	0.6631	0.8346	−0.1912	0.033*	
C7	0.7493 (10)	0.4348 (7)	−0.1073 (8)	0.0236 (19)	
C8	0.7618 (12)	0.3885 (6)	−0.2172 (8)	0.0201 (18)	
C9	0.8629 (11)	0.4246 (8)	−0.2909 (7)	0.0260 (18)	
H9	0.9222	0.4836	−0.2759	0.031*	
C10	0.8765 (15)	0.3728 (8)	−0.3881 (8)	0.037 (3)	
H10	0.9445	0.3957	−0.4409	0.044*	
C11	0.7875 (15)	0.2869 (8)	−0.4052 (9)	0.039 (3)	
H11	0.7936	0.2499	−0.4704	0.047*	
C12	0.6904 (13)	0.2556 (8)	−0.3268 (8)	0.032 (2)	
H12	0.6304	0.1966	−0.3398	0.038*	
C13	0.7855 (11)	0.4058 (6)	0.0817 (7)	0.0183 (18)	
C14	0.8309 (11)	0.3302 (7)	0.1689 (7)	0.0241 (18)	
C15	0.9449 (11)	0.3464 (6)	0.2615 (7)	0.026 (2)	
H15	0.9938	0.4083	0.2763	0.031*	
C16	0.9871 (12)	0.2702 (7)	0.3332 (8)	0.030 (2)	
H16	1.0673	0.2788	0.3980	0.036*	
C17	0.9129 (12)	0.1821 (7)	0.3106 (8)	0.029 (2)	
H17	0.9379	0.1296	0.3604	0.035*	
C18	0.8011 (12)	0.1713 (8)	0.2139 (9)	0.031 (2)	
H18	0.7514	0.1098	0.1976	0.037*	
C19	0.7492 (11)	0.5042 (6)	0.1010 (7)	0.0212 (18)	
C20	0.7376 (11)	0.5527 (6)	0.2092 (7)	0.0203 (18)	
C21	0.6956 (13)	0.5065 (8)	0.3028 (7)	0.031 (2)	
H21	0.6684	0.4399	0.3008	0.038*	
C22	0.6940 (13)	0.5593 (8)	0.3988 (8)	0.032 (2)	
H22	0.6659	0.5291	0.4640	0.039*	
C23	0.7325 (14)	0.6542 (8)	0.3998 (9)	0.037 (2)	
H23	0.7372	0.6902	0.4668	0.045*	
C24	0.7651 (12)	0.6983 (7)	0.3031 (8)	0.026 (2)	
H24	0.7877	0.7656	0.3038	0.032*	
I2	0.25693 (9)	0.48423 (5)	0.46473 (6)	0.0427 (2)	
O3	0.1465 (11)	0.5575 (6)	0.1752 (7)	0.053 (2)	
H3A	0.2127	0.5245	0.1425	0.079*	
H3B	0.1894	0.5500	0.2419	0.079*	

### Atomic displacement parameters (Å^2^)

**Table:**

	*U*^11^	*U*^22^	*U*^33^	*U*^12^	*U*^13^	*U*^23^
Mn1	0.0255 (7)	0.0152 (7)	0.0231 (7)	0.0003 (5)	0.0012 (5)	−0.0020 (5)
I1	0.0437 (4)	0.0208 (3)	0.0499 (4)	−0.0005 (3)	0.0033 (3)	−0.0050 (3)
O1	0.023 (3)	0.029 (4)	0.046 (4)	0.002 (3)	0.002 (3)	0.015 (3)
O2	0.021 (3)	0.033 (4)	0.026 (3)	0.002 (3)	0.004 (3)	−0.003 (3)
N1	0.025 (4)	0.012 (3)	0.019 (4)	−0.007 (3)	0.000 (3)	−0.007 (3)
N2	0.021 (4)	0.024 (4)	0.025 (4)	0.005 (3)	0.003 (3)	−0.005 (3)
N3	0.027 (4)	0.010 (3)	0.026 (4)	0.000 (3)	0.006 (3)	−0.002 (3)
N4	0.025 (4)	0.023 (4)	0.028 (4)	0.002 (3)	0.011 (3)	−0.010 (3)
N5	0.029 (4)	0.019 (4)	0.026 (4)	0.002 (3)	0.007 (3)	0.001 (3)
N6	0.035 (5)	0.023 (4)	0.012 (3)	0.006 (3)	−0.002 (3)	−0.001 (3)
C1	0.020 (4)	0.015 (4)	0.022 (4)	0.004 (3)	−0.002 (3)	−0.006 (3)
C2	0.033 (5)	0.022 (4)	0.012 (4)	−0.001 (4)	−0.003 (4)	−0.002 (3)
C3	0.029 (5)	0.025 (5)	0.027 (5)	0.004 (4)	0.008 (4)	−0.002 (4)
C4	0.032 (5)	0.022 (5)	0.031 (5)	0.001 (4)	0.006 (4)	−0.006 (4)
C5	0.032 (5)	0.035 (5)	0.008 (4)	0.005 (4)	0.008 (3)	0.000 (3)
C6	0.032 (5)	0.015 (4)	0.033 (5)	0.003 (4)	0.000 (4)	−0.002 (4)
C7	0.017 (4)	0.018 (5)	0.035 (5)	0.001 (4)	0.001 (3)	−0.002 (4)
C8	0.024 (5)	0.013 (4)	0.026 (5)	0.005 (3)	0.012 (4)	−0.001 (3)
C9	0.036 (5)	0.015 (4)	0.028 (4)	−0.006 (4)	0.008 (4)	0.001 (4)
C10	0.051 (7)	0.045 (6)	0.014 (4)	0.004 (5)	0.005 (4)	0.001 (4)
C11	0.054 (7)	0.038 (6)	0.025 (5)	0.010 (5)	0.002 (5)	−0.013 (5)
C12	0.043 (6)	0.033 (5)	0.021 (5)	−0.003 (5)	0.009 (4)	−0.007 (4)
C13	0.023 (4)	0.009 (4)	0.021 (4)	−0.001 (3)	−0.002 (3)	−0.001 (3)
C14	0.028 (4)	0.020 (4)	0.022 (4)	0.002 (4)	−0.003 (3)	0.000 (4)
C15	0.022 (4)	0.021 (5)	0.032 (5)	−0.001 (3)	−0.007 (4)	−0.005 (4)
C16	0.031 (5)	0.024 (5)	0.032 (5)	0.004 (4)	−0.004 (4)	−0.004 (4)
C17	0.031 (5)	0.032 (5)	0.026 (5)	0.010 (4)	0.008 (4)	0.007 (4)
C18	0.028 (5)	0.029 (5)	0.039 (6)	0.001 (4)	0.011 (4)	0.005 (4)
C19	0.023 (4)	0.019 (5)	0.021 (4)	−0.003 (3)	0.004 (3)	−0.006 (3)
C20	0.019 (4)	0.019 (4)	0.026 (5)	0.000 (3)	0.011 (3)	0.002 (4)
C21	0.044 (6)	0.033 (6)	0.017 (4)	−0.005 (5)	0.007 (4)	0.000 (4)
C22	0.040 (6)	0.038 (6)	0.022 (5)	0.002 (5)	0.011 (4)	0.009 (4)
C23	0.045 (6)	0.038 (6)	0.027 (5)	0.002 (5)	−0.001 (5)	−0.010 (4)
C24	0.033 (5)	0.017 (5)	0.030 (5)	−0.005 (4)	0.010 (4)	−0.003 (4)
I2	0.0438 (4)	0.0399 (4)	0.0443 (4)	−0.0125 (4)	0.0054 (3)	−0.0141 (4)
O3	0.059 (6)	0.046 (5)	0.053 (5)	0.013 (4)	0.009 (4)	0.013 (4)

### Geometric parameters (Å, °)

**Table:**

Mn1—O1	2.163 (6)	C6—H6	0.9500
Mn1—O2	2.182 (6)	C7—C8	1.497 (13)
Mn1—N6	2.245 (7)	C8—C9	1.378 (12)
Mn1—N1	2.253 (7)	C9—C10	1.399 (13)
Mn1—N3	2.259 (7)	C9—H9	0.9500
Mn1—I1	2.7772 (16)	C10—C11	1.389 (15)
O1—H1A	0.8400	C10—H10	0.9500
O1—H1B	0.8400	C11—C12	1.377 (15)
O2—H2A	0.8400	C11—H11	0.9500
O2—H2B	0.8400	C12—H12	0.9500
N1—C1	1.328 (10)	C13—C19	1.423 (12)
N1—C19	1.350 (11)	C13—C14	1.498 (12)
N2—C13	1.314 (11)	C14—C15	1.362 (11)
N2—C7	1.334 (11)	C15—C16	1.382 (13)
N3—C6	1.346 (11)	C15—H15	0.9500
N3—C2	1.358 (11)	C16—C17	1.369 (13)
N4—C12	1.339 (11)	C16—H16	0.9500
N4—C8	1.356 (11)	C17—C18	1.380 (13)
N5—C18	1.347 (12)	C17—H17	0.9500
N5—C14	1.351 (12)	C18—H18	0.9500
N6—C24	1.330 (11)	C19—C20	1.489 (12)
N6—C20	1.363 (11)	C20—C21	1.385 (12)
C1—C7	1.387 (11)	C21—C22	1.377 (13)
C1—C2	1.525 (12)	C21—H21	0.9500
C2—C3	1.349 (12)	C22—C23	1.352 (15)
C3—C4	1.385 (13)	C22—H22	0.9500
C3—H3	0.9500	C23—C24	1.381 (14)
C4—C5	1.385 (13)	C23—H23	0.9500
C4—H4	0.9500	C24—H24	0.9500
C5—C6	1.381 (12)	O3—H3A	0.8400
C5—H5	0.9500	O3—H3B	0.8400
			
O1—Mn1—O2	167.0 (2)	C1—C7—C8	126.0 (8)
O1—Mn1—N6	85.5 (3)	N4—C8—C9	123.4 (8)
O2—Mn1—N6	83.0 (3)	N4—C8—C7	113.8 (7)
O1—Mn1—N1	87.4 (3)	C9—C8—C7	122.6 (8)
O2—Mn1—N1	83.3 (3)	C8—C9—C10	118.7 (10)
N6—Mn1—N1	71.7 (3)	C8—C9—H9	120.6
O1—Mn1—N3	94.1 (3)	C10—C9—H9	120.6
O2—Mn1—N3	91.5 (2)	C11—C10—C9	118.0 (10)
N6—Mn1—N3	143.1 (3)	C11—C10—H10	121.0
N1—Mn1—N3	71.4 (2)	C9—C10—H10	121.0
O1—Mn1—I1	96.54 (18)	C12—C11—C10	119.5 (9)
O2—Mn1—I1	93.67 (17)	C12—C11—H11	120.3
N6—Mn1—I1	114.2 (2)	C10—C11—H11	120.3
N1—Mn1—I1	173.04 (19)	N4—C12—C11	123.3 (10)
N3—Mn1—I1	102.48 (18)	N4—C12—H12	118.4
Mn1—O1—H1A	116.2	C11—C12—H12	118.4
Mn1—O1—H1B	121.2	N2—C13—C19	119.3 (8)
H1A—O1—H1B	119.7	N2—C13—C14	114.4 (7)
Mn1—O2—H2A	118.3	C19—C13—C14	126.2 (8)
Mn1—O2—H2B	102.7	N5—C14—C15	124.1 (9)
H2A—O2—H2B	116.6	N5—C14—C13	113.3 (7)
C1—N1—C19	120.6 (7)	C15—C14—C13	122.4 (8)
C1—N1—Mn1	119.2 (6)	C14—C15—C16	118.0 (8)
C19—N1—Mn1	118.6 (5)	C14—C15—H15	121.0
C13—N2—C7	121.2 (8)	C16—C15—H15	121.0
C6—N3—C2	117.4 (7)	C17—C16—C15	119.8 (8)
C6—N3—Mn1	121.8 (6)	C17—C16—H16	120.1
C2—N3—Mn1	118.5 (5)	C15—C16—H16	120.1
C12—N4—C8	117.1 (8)	C16—C17—C18	118.6 (9)
C18—N5—C14	116.4 (8)	C16—C17—H17	120.7
C24—N6—C20	118.6 (8)	C18—C17—H17	120.7
C24—N6—Mn1	121.5 (6)	N5—C18—C17	123.1 (10)
C20—N6—Mn1	117.5 (6)	N5—C18—H18	118.5
N1—C1—C7	119.7 (8)	C17—C18—H18	118.5
N1—C1—C2	113.3 (7)	N1—C19—C13	118.8 (8)
C7—C1—C2	126.9 (8)	N1—C19—C20	113.1 (7)
C3—C2—N3	123.2 (8)	C13—C19—C20	128.1 (8)
C3—C2—C1	123.5 (8)	N6—C20—C21	121.2 (8)
N3—C2—C1	113.0 (7)	N6—C20—C19	114.3 (7)
C2—C3—C4	118.9 (9)	C21—C20—C19	124.5 (8)
C2—C3—H3	120.5	C22—C21—C20	118.6 (9)
C4—C3—H3	120.5	C22—C21—H21	120.7
C3—C4—C5	119.2 (9)	C20—C21—H21	120.7
C3—C4—H4	120.4	C23—C22—C21	119.8 (9)
C5—C4—H4	120.4	C23—C22—H22	120.1
C6—C5—C4	118.4 (8)	C21—C22—H22	120.1
C6—C5—H5	120.8	C22—C23—C24	119.6 (9)
C4—C5—H5	120.8	C22—C23—H23	120.2
N3—C6—C5	122.5 (8)	C24—C23—H23	120.2
N3—C6—H6	118.8	N6—C24—C23	121.9 (9)
C5—C6—H6	118.8	N6—C24—H24	119.0
N2—C7—C1	120.2 (9)	C23—C24—H24	119.0
N2—C7—C8	113.7 (8)	H3A—O3—H3B	100.9
			
O1—Mn1—N1—C1	−86.0 (7)	N1—C1—C7—C8	−175.3 (8)
O2—Mn1—N1—C1	103.1 (7)	C2—C1—C7—C8	8.2 (15)
N6—Mn1—N1—C1	−172.1 (7)	C12—N4—C8—C9	−1.2 (13)
N3—Mn1—N1—C1	9.2 (6)	C12—N4—C8—C7	−176.0 (8)
O1—Mn1—N1—C19	79.9 (6)	N2—C7—C8—N4	51.0 (10)
O2—Mn1—N1—C19	−91.0 (6)	C1—C7—C8—N4	−133.2 (9)
N6—Mn1—N1—C19	−6.2 (6)	N2—C7—C8—C9	−123.8 (10)
N3—Mn1—N1—C19	175.1 (7)	C1—C7—C8—C9	52.0 (14)
O1—Mn1—N3—C6	−106.4 (7)	N4—C8—C9—C10	1.1 (14)
O2—Mn1—N3—C6	85.3 (7)	C7—C8—C9—C10	175.4 (9)
N6—Mn1—N3—C6	165.7 (6)	C8—C9—C10—C11	−0.3 (15)
N1—Mn1—N3—C6	167.8 (7)	C9—C10—C11—C12	−0.2 (15)
I1—Mn1—N3—C6	−8.8 (7)	C8—N4—C12—C11	0.7 (15)
O1—Mn1—N3—C2	91.2 (7)	C10—C11—C12—N4	0.0 (16)
O2—Mn1—N3—C2	−77.0 (7)	C7—N2—C13—C19	−2.5 (13)
N6—Mn1—N3—C2	3.3 (9)	C7—N2—C13—C14	180.0 (8)
N1—Mn1—N3—C2	5.4 (6)	C18—N5—C14—C15	2.3 (14)
I1—Mn1—N3—C2	−171.1 (6)	C18—N5—C14—C13	176.9 (8)
O1—Mn1—N6—C24	101.1 (7)	N2—C13—C14—N5	−42.3 (11)
O2—Mn1—N6—C24	−85.0 (7)	C19—C13—C14—N5	140.4 (9)
N1—Mn1—N6—C24	−170.2 (8)	N2—C13—C14—C15	132.4 (9)
N3—Mn1—N6—C24	−168.2 (6)	C19—C13—C14—C15	−44.9 (14)
I1—Mn1—N6—C24	5.9 (8)	N5—C14—C15—C16	−1.4 (15)
O1—Mn1—N6—C20	−97.0 (7)	C13—C14—C15—C16	−175.6 (9)
O2—Mn1—N6—C20	77.0 (7)	C14—C15—C16—C17	−0.9 (14)
N1—Mn1—N6—C20	−8.3 (6)	C15—C16—C17—C18	2.1 (14)
N3—Mn1—N6—C20	−6.2 (9)	C14—N5—C18—C17	−0.9 (14)
I1—Mn1—N6—C20	167.9 (6)	C16—C17—C18—N5	−1.2 (15)
C19—N1—C1—C7	−3.1 (13)	C1—N1—C19—C13	3.1 (12)
Mn1—N1—C1—C7	162.5 (6)	Mn1—N1—C19—C13	−162.6 (6)
C19—N1—C1—C2	173.9 (8)	C1—N1—C19—C20	−176.1 (8)
Mn1—N1—C1—C2	−20.5 (10)	Mn1—N1—C19—C20	18.2 (9)
C6—N3—C2—C3	−5.4 (14)	N2—C13—C19—N1	−0.3 (13)
Mn1—N3—C2—C3	157.7 (8)	C14—C13—C19—N1	176.9 (8)
C6—N3—C2—C1	179.9 (8)	N2—C13—C19—C20	178.7 (8)
Mn1—N3—C2—C1	−17.0 (10)	C14—C13—C19—C20	−4.0 (15)
N1—C1—C2—C3	−150.6 (9)	C24—N6—C20—C21	5.2 (13)
C7—C1—C2—C3	26.1 (15)	Mn1—N6—C20—C21	−157.3 (7)
N1—C1—C2—N3	24.1 (11)	C24—N6—C20—C19	−177.2 (8)
C7—C1—C2—N3	−159.2 (9)	Mn1—N6—C20—C19	20.3 (10)
N3—C2—C3—C4	5.7 (15)	N1—C19—C20—N6	−24.9 (11)
C1—C2—C3—C4	179.8 (9)	C13—C19—C20—N6	156.0 (9)
C2—C3—C4—C5	−1.2 (14)	N1—C19—C20—C21	152.6 (9)
C3—C4—C5—C6	−3.3 (13)	C13—C19—C20—C21	−26.5 (15)
C2—N3—C6—C5	0.6 (13)	N6—C20—C21—C22	−4.5 (15)
Mn1—N3—C6—C5	−161.9 (7)	C19—C20—C21—C22	178.2 (9)
C4—C5—C6—N3	3.6 (14)	C20—C21—C22—C23	0.2 (16)
C13—N2—C7—C1	2.6 (13)	C21—C22—C23—C24	3.2 (16)
C13—N2—C7—C8	178.6 (8)	C20—N6—C24—C23	−1.7 (14)
N1—C1—C7—N2	0.3 (13)	Mn1—N6—C24—C23	160.1 (8)
C2—C1—C7—N2	−176.2 (8)	C22—C23—C24—N6	−2.5 (16)

### Hydrogen-bond geometry (Å, °)

**Table:**

*D*—H···*A*	*D*—H	H···*A*	*D*···*A*	*D*—H···*A*
O1—H1A···O3^i^	0.84	1.92	2.726 (10)	160.
O1—H1B···N2^ii^	0.84	2.10	2.890 (10)	156.
O2—H2A···N4^iii^	0.84	2.04	2.876 (10)	174.
O2—H2B···N5^iii^	0.84	2.03	2.834 (10)	160.
O3—H3A···I1^iv^	0.84	2.88	3.615 (9)	148.
O3—H3B···I2	0.84	2.83	3.646 (8)	163.

Symmetry codes: (i) *x*+1, *y*, *z*; (ii) −*x*+2, *y*+1/2, −*z*; (iii) −*x*+1, *y*+1/2, −*z*; (iv) −*x*+1, *y*−1/2, −*z*.

## References

[bb1] Bruker (2000). *SADABS*, *SMART* and *SAINT* Bruker AXS Inc., Madison, Wisconsin, USA.

[bb2] Callejo, L., De la Pinta, N., Vitoria, P. & Cortés, R. (2009). *Acta Cryst.* E**65**, m68–m69. 10.1107/S1600536808041755 PMC2967907 21581535

[bb3] Farrugia, L. J. (1997). *J. Appl. Cryst.* **30**, 565.

[bb4] Flack, H. D. (1983). *Acta Cryst.* A**39**, 876–881.

[bb5] Ha, K. (2011). *Z. Kristallogr. New Cryst. Struct.* **226**, 59–60.

[bb6] Sheldrick, G. M. (2008). *Acta Cryst.* A**64**, 112–122. 10.1107/S0108767307043930 18156677

[bb7] Spek, A. L. (2009). *Acta Cryst.* D**65**, 148–155. 10.1107/S090744490804362X PMC2631630 19171970

